# Effectiveness of financial incentives for control of viral hepatitis among substance users: a systematic review and meta-analysis

**DOI:** 10.3389/fpubh.2024.1394164

**Published:** 2024-11-14

**Authors:** Wanchen Wang, Lu Zhang

**Affiliations:** ^1^School of Public Health, Shandong Second Medical University, Weifang, Shandong, China; ^2^Centre for Health Management and Policy Research, School of Public Health, Cheeloo College of Medicine, Shandong University, Jinan, Shandong, China; ^3^Jinan Medical Technology Carefree Medical Research Institute, Jinan, Shandong, China

**Keywords:** hepatitis, incentives, meta-analysis, substance abuse, systematic review

## Abstract

**Background:**

Hepatitis B virus (HBV) poses a significant global health challenge in substance users who are at a higher risk of infection. Financial incentives have been proposed as a strategy to enhance vaccine uptake among high-risk groups. This meta-analysis aims to assess the effectiveness of financial incentives in increasing HBV vaccination rates among substance users.

**Methods:**

A literature search across various databases was done for randomized controlled trials (RCTs) and non-randomized trials evaluating the impact of financial incentives on HBV vaccination rates in substance users. Six studies with a total of 3,886 participants were included. The GRADE approach was used to assess the quality of evidence, and a random-effects meta-analysis was done to calculate pooled risk ratios (RRs) for vaccination uptake.

**Results:**

Financial incentives were associated with a significant increase in the HBV vaccination uptake rates among substance users, with pooled RR of 2.261 (95% CI: 1.327–3.851), despite considerable heterogeneity (I^2^ = 93.7%). Sensitivity analysis confirmed the robustness of these findings. However, GRADE assessment indicated a very low quality of evidence, primarily due to risk of bias, inconsistency, imprecision, and potential publication bias, highlighted by a significant Luis Furuya–Kanamori (LFK) index of 6.42.

**Conclusion:**

Financial incentives significantly improve HBV vaccination rates among substance users, underscoring their potential as a public health intervention in this high-risk population. Low quality of evidence calls for further high-quality RCTs to confirm these results and explore the most effective incentive strategies.

**Systematic review registration:**

https://www.crd.york.ac.uk/prospero/display_record.php?ID=CRD42024505277, identifier CRD42024505277.

## Introduction

Viral hepatitis, especially hepatitis B (HBV), represents a significant global public health challenge, particularly among populations with high-risk behaviors such as substance users (1). The World Health Organization (WHO) identifies viral hepatitis as a leading cause of liver disease and mortality worldwide (2). HBV infections are particularly prevalent among substance users due to behaviors such as the sharing of needles and other drug paraphernalia, which significantly increase the risk of transmission (3). Since HBV is associated with substantial morbidity, mortality, and socioeconomic burden, there is a pressing need for effective strategies to control its spread within high-risk populations (4).

Substance users face numerous barriers to accessing healthcare services, including stigma, lack of awareness, financial constraints, and the transient nature of this population (5, 6). As a result, rates of hepatitis testing, vaccination, and treatment uptake among substance users is significantly lower compared to the general population (7, 8). Therefore, innovative approaches, such as financial incentives, may potentially increase the participation of substance users in hepatitis prevention and treatment programs (9). Financial incentives, including cash or vouchers, are provided to individuals as a reward for engaging in health-promoting behaviors, like completing vaccination series (10–12). The general idea is that such incentives can motivate behavior change by providing a tangible reward for actions that these individuals might otherwise neglect due to various barriers (10, 13).

The concept of using financial incentives to influence health behaviors is supported by theories of behavioral economics, which suggest that individuals are more likely to engage in health-promoting behaviors when provided with immediate rewards (10, 14). Nevertheless, despite the potential of financial incentives to improve health outcomes, their effectiveness in controlling HBV in substance users is still unclear. While some studies have reported positive outcomes, including increased rates of vaccination, others have found limited or no impact (15–17). This review aims to assess the value of financial incentives in improving the uptake of HBV vaccination among substance users.

## Methods

### Eligibility criteria

Population: We included studies conducted on patients who are current substance users, defined as individuals actively using substances at the time of the study. Studies focusing on other populations, such as former substance users or those not using substances, were excluded.

Intervention: The intervention of interest was the provision of financial incentives aimed at increasing HBV vaccination rates. Financial incentives could include cash payments, vouchers, or other monetary rewards given to participants for receiving the HBV vaccine. Studies needed to clearly define the type, amount, and delivery method of the financial incentives to be included in the analysis.

Comparison: The comparator was the usual care arm, which included standard practices for encouraging HBV vaccination without additional financial incentives. Usual care could involve educational interventions, reminders, or other non-monetary methods.

Outcome: The primary outcome of interest was HBV vaccination coverage, defined as the proportion of the target population that received one or more doses of the HBV vaccine.

Study Design: We included parallel-arm individual randomized controlled trials (RCTs), cluster RCTs, and non-RCTs.

Publication status: Only full-text studies published in peer-reviewed journals were included to ensure the reliability and validity of the findings. Studies needed to provide sufficient methodological detail to allow for quality assessment and data extraction. Abstracts, conference proceedings, and unpublished data were excluded to avoid the inclusion of incomplete or non-peer-reviewed information. Additionally, studies published in languages other than English were excluded due to resource limitations for translation.

### Information sources

Through search was conducted in Medline Ovid, Scopus, EMBASE, Cochrane library, ClinicalTrials.gov, and the WHO trials registries.

### Search strategy

Terms such as “Hepatitis B,” “Financial Incentives,” “Conditional Cash Transfer,” “Randomized Controlled Trial,” and “Hepatitis B vaccine” were utilized in various combinations across all the databases mentioned, from their inception until January 2024, with no publication language restrictions. Detailed search for each of the databases are provided in Supplementary file 1. The search strategy was designed to increase the sensitivity and comprehensiveness by including a broader range of synonyms and relevant terms for each concept (Hepatitis B, financial incentives, and study design). By incorporating both controlled vocabulary (MeSH terms) and free-text terms, the strategy aims to capture all relevant studies, including those that might not use standard terminology.

Reference lists of retrieved studies were then manually searched for additional relevant articles. Study authors were contacted in cases where clarification or additional information was required. Two authors (WW and LZ) independently conducted the search.

### Selection process

The study selection process was also conducted independently by two investigators (WW and LZ). Titles and abstracts of all identified studies were searched for possible inclusion, and full-texts of relevant articles were the assessed independently by primary and secondary investigators for eligibility (WW and LZ). All disagreements were resolved through consensus.

Data collection process.

General information, methods section containing design, details of the participants, and setting, total sample in each group, baseline, endline values, and criteria, interventions related details, and outcomes was extracted. Data related to outcome measures were independently extracted by primary and secondary investigators. In case of studies with multiple arms in a single trial, only the relevant arms were included in the analysis.

### Study risk of bias assessment

Study quality was assessed by two reviewers using the Cochrane Collaboration’s Risk of Bias 2 (RoB-2) tool for RCTs (18), and the risk of bias tool for non-randomized trials (ROBINS-I) (19). Based on this assessment, studies were classified as ‘low,’ ‘high,’ or ‘some concerns’ in terms of the bias risk.

### Effect measures and synthesis methods

STATA software, version 14.2 was used for analysis. Given that the data were dichotomous, the risk ratio (RR) and its 95% confidence interval (CI) were calculated based on the frequency of events observed in the intervention and control groups, offering a comparative assessment of the intervention effects.

To accommodate the variability across studies, a random-effects model was applied, using the inverse variance method (20). Heterogeneity was assessed by the inspection of confidence interval overlaps in forest plots, chi-square tests, and by I^2^ statistic (20). A sensitivity analysis was carried out to determine the impact of individual studies on the overall results.

### Reporting bias assessment

Due to the smaller number of studies (less than 10), traditional methods for publication bias analysis, like Egger’s test and funnel plots, were not feasible. The Doi plot and the Luis Furuya Kanamori (LFK) index were used as alternative approaches to explore and quantify potential publication bias (21). The LFK index ranges from-1 to +1, indicating no publication bias (perfect symmetry). Values between-1 to-2 or + 1 to +2 suggest minor asymmetry, while values less than-2 or greater than +2 indicate major asymmetry.

### Certainty assessment

Grading of Recommendations Assessment, Development, and Evaluation (GRADE) involves a systematic evaluation of the quality of evidence across several domains (22). This includes:

Risk of Bias: Potential biases that could affect the validity of the findings were assessed. The Cochrane risk of bias tools were used for this purpose.

Inconsistency: Examination of heterogeneity across study results, including statistical measures such as I^2^ and Cochran’s Q, to assess variations in effect sizes.

Indirectness: Evaluation of the directness of the evidence in addressing the research question, including the applicability of the study populations, interventions, and outcomes to the context of interest.

Imprecision: Analysis of the confidence intervals around the effect estimates to determine the certainty of the findings.

Publication Bias: Investigation of the potential for publication bias, using statistical tools like the LFK index, to identify asymmetry in the meta-analysis that could indicate missing studies or small study effects.

Based on these domains, we classified the quality of evidence into four levels: high, moderate, low, or very low. These levels reflect our confidence in the effect estimate: the higher the quality, the more likely it is that the true effect lies close to the estimate of the effect.

## Results

### Study selection

**Figure 1:**
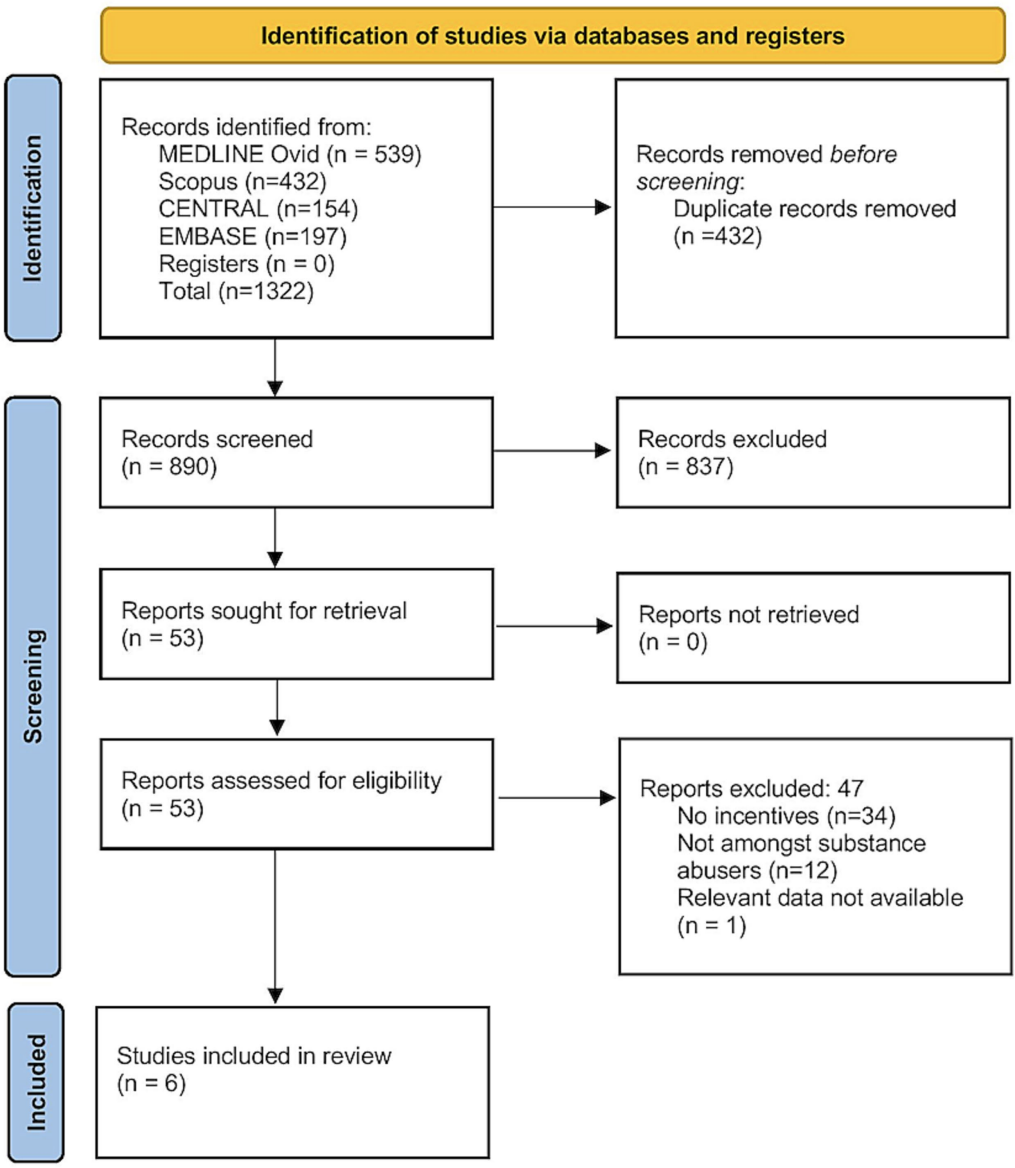
PRISMA flowchart.

A total of 1,322 records were retrieved from all the databases. Of them, 890 records remained after deduplication, and underwent primary screening. Full-texts of 53 studies were screened for eligibility, and finally, six studies were included in the analysis (Figure 1) (15–17, 23–25).

### Study characteristics

As shown in Table 1, all six studies reported the efficacy of financial incentives in promoting hepatitis B vaccination among substance users. Of them, five were RCTs and one was a non-randomized trial. Studies were done in the United States, Australia, and the United Kingdom. Participant age ranged from 18 to 65 years, and the sample sizes varied from 13 to 1,158 in the intervention arms, and from 13 to 2023 in the control arms. The interventions involved monetary incentives of varying amounts and forms, aiming to enhance vaccination uptake. Gender distribution across studies showed a higher prevalence of male participants.

**Table 1:** Characteristics of the included studies in the meta-analysis.

Author and year	Study design	Location	Study participants	Sample size	Outcome details	Intervention details	Usual care details	Gender distribution	Age in years
Stitzer et al. 2009	Randomized Controlled Trial	United States	Participants included aged 18–64 years, meets diagnostic criteria for cocaine abuse or dependence, agrees to a 6-month regimen of the HBV vaccine, and reads English.	I = 13\u00B0C = 13	Completed the vaccination schedule	Participants are randomly assigned to incentive or control conditions and expected to meet with research staff for 1 h each week for 24 weeks. Maximum incentives that can be earned in intervention arm is $751 and $20 for completing study procedures	Usual care participants received only $20 for completing study procedures	21 Males and 5 Females	Average age was 45 years. Incentive (mean = 48) and control (mean age = 41, SD = 11.7)
Topp et al. 2013	Randomized Controlled Trial	Australia	Participants aged 16 years and above and injected drugs in the preceding 6 months with no previous HBV infection and a maximum of one previous vaccination dose, or unknown infection and vaccination status and willing to be randomized, to undertake vaccination, and to attend follow-up 12 weeks post-randomization.	I = 74\u00B0C = 65	Completed the vaccination schedule	$30 Australian Dollars cash following receipt of vaccine doses two and three (‘incentive condition’) and $20 shopping voucher for study completion	$20 shopping voucher for study participation only	107 males and 32 females	Mean age of 33.1 years (SD 8.4)
Weaver et al. 2014	Cluster randomized trial	United Kingdom	Participants with previous, current, or future risk of injecting drug use and agreed to receive vaccination, participate in the trial, and provided written informed consent.	I = 143\u00B0C = 67	Completion of vaccination schedule within 28 days	Escalating value contingency management (£5, £10, and £15 vouchers)	Offered vaccination without any incentive	167 males and 43 females	18–65 years
Trubatch et al. 2000	Non-randomized trial	United States	Street-recruited IDUs who are participating in a National Institute on Drug Abuse–funded study are offered hepatitis B vaccination	I = 172\u00B0C = 140	Receipt of first Hepatitis B vaccination	Monetary incentive of $10 in the incentive arm	No incentive and treatment as usual	Not mentioned	Not mentioned
Campbell et al. 2007	Randomized Controlled Trial	United States	Those who injected drugs in the past 6 months, willing to provide locator information and a blood specimen for serologic testing, spoke English and had no plans to move in the following 12 months	I = 1,158\u00B0C = 2023	Receipt of one or more dose of Hepatitis B vaccine	Participants received standardized HIV and viral hepatitis pre-test counseling, and were offered free vaccination, on a flexible 0-, 1-, 6-month schedule and monetary incentives of $5 per dose	Treatment as usual without incentive	Not mentioned	18–30 years
Seal et al. 2003	Randomized Controlled Trial	United States	Those who lacked all three HBV seromarkers and those with antibodies to HBV core antigen (anti-HBc) only were offered enrolment.	I = 48\u00B0C = 48	Complete vaccination schedule	Participants were randomized to either the monetary incentive or outreach arms and received the first dose of hepatitis B. Monetary incentive arm received a modest cash incentive ($20) each month for 6 months.	Maintain weekly contact with outreach worker	69 males and 27 females	Mean age = 43 Years

### Risk of bias in studies

Among the five RCTs, all of them were assessed to have a low risk with respect to randomization domain. Confounding was assessed in one non-RCT showed some concerns, while participant selection indicating high risk and classification of intervention had lower risk of bias. For deviation from the intended intervention, four studies had low risk, two had some concerns. Missing outcome data was low risk in three studies and high risk in three studies. Outcome measurement showed low risk in three studies, high risk in two studies and some concerns in one study. Selective outcome reporting was low risk in one study, some concerns in one study, and high risk in four studies. Two studies had a high risk of bias, one study had a low risk of bias, and the remaining studies had some concerns or not specified (Table 2).

**Table 2:** Risk of bias assessment.

Author and year	Randomization process	Confounding	Participant selection	Classification of intervention	Deviation from intended intervention	Missing outcome data	Outcome measurement	Selective outcome reporting	Risk of bias
Stitzer et al. 2009	Low	NA	NA	NA	Low	Low	Low	Some concerns	Some concerns
Topp et al. 2013	Low	NA	NA	NA	Some concerns	High	Low	Some concerns	High
Weaver et al. 2014	Low	NA	NA	NA	Low	Low	Low	Low	Low
Trubatch et al. 2000	NA	Some concerns	High	Low	Low	Low	Some concerns	Some concerns	High
Campbell et al. 2007	Low	NA	NA	NA	Some concerns	High	High	High	High
Seal et al. 2003	Low	NA	NA	NA	Low	High	High	Some concerns	High

NA, not applicable.

### Results of individual studies

The individual studies included in this review present a comprehensive analysis of financial incentives on hepatitis B vaccination uptake among substance users. Seal et al. (2003) conducted a randomized controlled trial comparing monetary incentives to outreach methods for hepatitis B vaccine adherence in IDUs (15). They found that 69% of participants in the incentive group completed the vaccine series compared to only 23% in the outreach group, demonstrating a significant positive effect of monetary incentives on vaccine adherence. Trubatch et al. reported that offering monetary incentives to IDUs in Anchorage, Alaska significantly increased hepatitis B vaccination rates, with 48% of incentivized participants receiving their first dose compared to 7% without incentives (16). Similarly, Stitzer et al. showed that prize-based incentives improved adherence to a 6-month hepatitis B vaccination protocol among cocaine users, with 74% of injections received on schedule in the incentive group compared to 51% in the control group (23). Campbell et al. highlighted the effectiveness of financial incentives in promoting health behaviors, showing substantial improvements in vaccination rates and suggesting a scalable approach for public health interventions (17). Topp et al. further corroborated these findings by demonstrating that incentivized participants had significantly higher vaccination uptake rates (24). Finally, Weaver et al. underscored the importance of tailored incentive programs to address the specific needs and barriers faced by substance users, enhancing overall public health outcomes (25). Together, these studies underscore the robust impact of financial incentives on improving hepatitis B vaccination rates among high-risk populations, suggesting their potential utility in broader public health strategies.

### Results of synthesis

#### Hepatitis vaccination coverage

**Figure 2:**
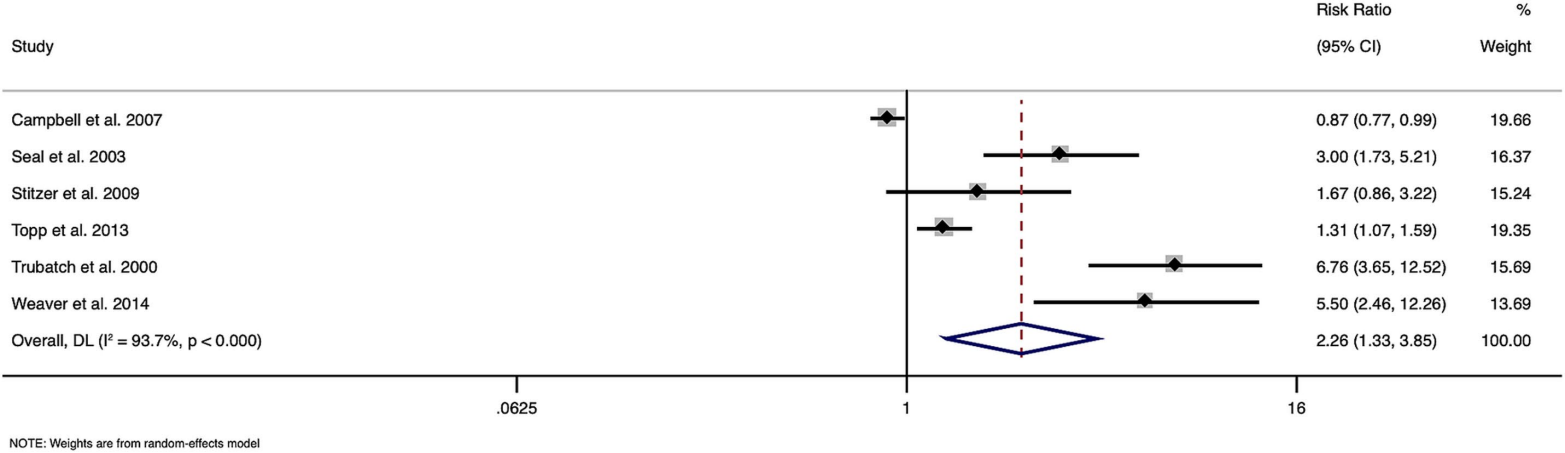
Forest plot showing the effectiveness of financial incentives for improving hepatitis vaccination coverage.

The meta-analysis of data from six papers with a total of 3,886 participants, showed an overall pooled RR of 2.261 (95% CI: 1.327 to 3.851), indicating a significant effect of financial incentives on hepatitis B vaccination uptake among substance users (Figure 2). Heterogeneity across studies was high (I^2^ = 93.7%, Cochran’s Q = 79.48, *p* < 0.0001), underscoring considerable variability in study outcomes. The overall effect test was statistically significant (z = 3.002, *p* = 0.003), supporting the effectiveness of financial incentives in improving vaccination rates.

Subgroup analysis based on type of outcome shows that the pooled RR for the single dose outcome was 2.372 (95% CI: 0.319–17.618, *p* = 0.398), and for completion of the vaccination schedule, it was 2.299 (95% CI: 1.233–4.289, *p* = 0.009; Supplementary Figure 1). Between-group heterogeneity was not significant (*p* = 0.977), indicating no significant difference in the effect sizes between these two outcome types.

Subgroup analysis also examined two types of incentives: incentive for each dose or regular incentive (RR: 1.582, 95% CI: 0.690–3.630, *p* = 0.279) and different incentive pattern (RR: 3.526, 95% CI: 1.018–12.220, *p* = 0.047). Between-group heterogeneity was not statistically significant (*p* = 0.293), suggesting that the type of incentive did not result in significantly different effects on vaccination uptake (Supplementary Figure 2).

**Figure 3:**
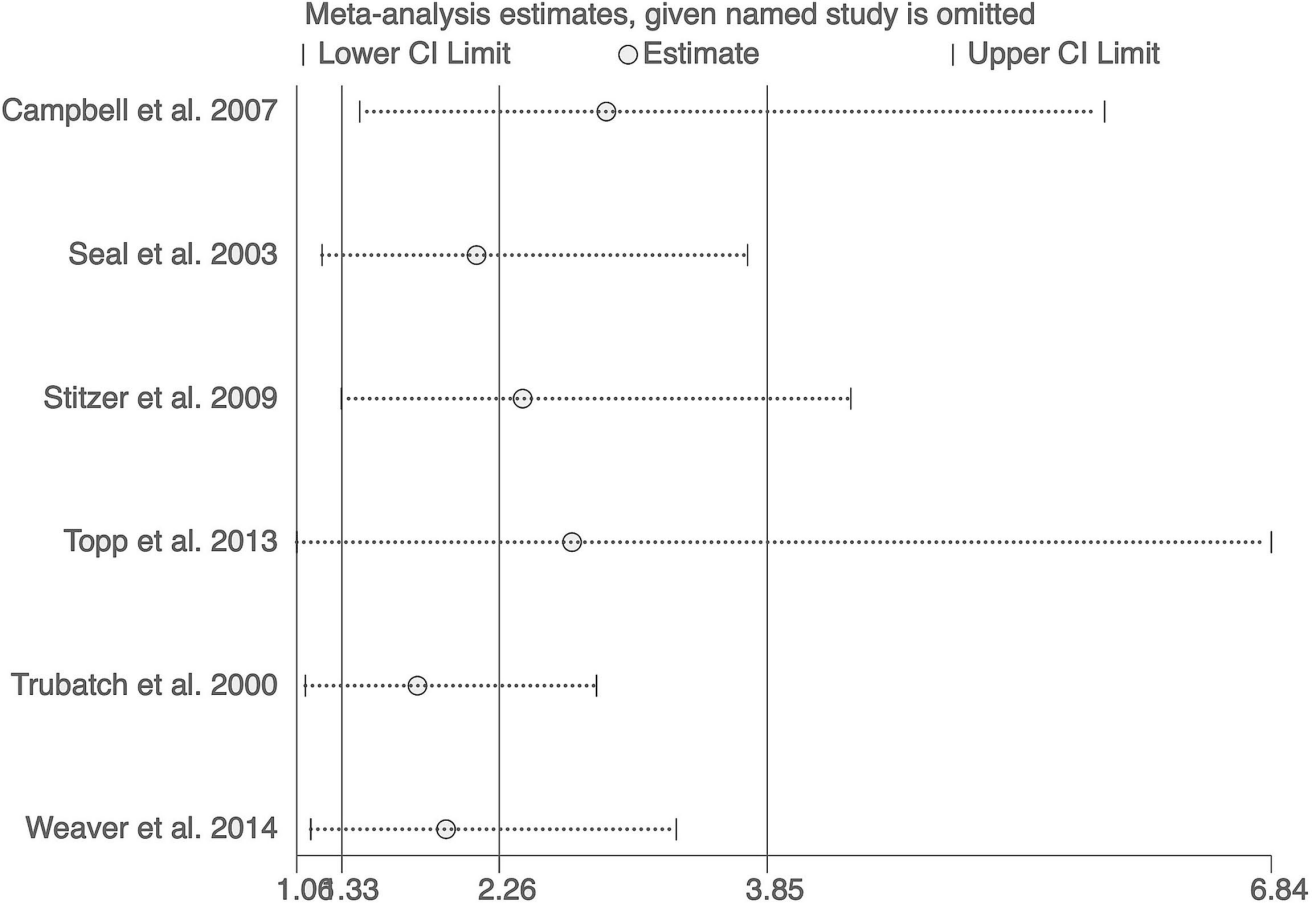
Sensitivity analysis plot.

The sensitivity analysis, excluding one study at a time, yielded combined estimates ranging from 1.77 to 2.90, consistently supporting the effectiveness of financial incentives in increasing hepatitis B vaccination rates among substance users (Figure 3).

#### Reporting biases

**Figure 4:**
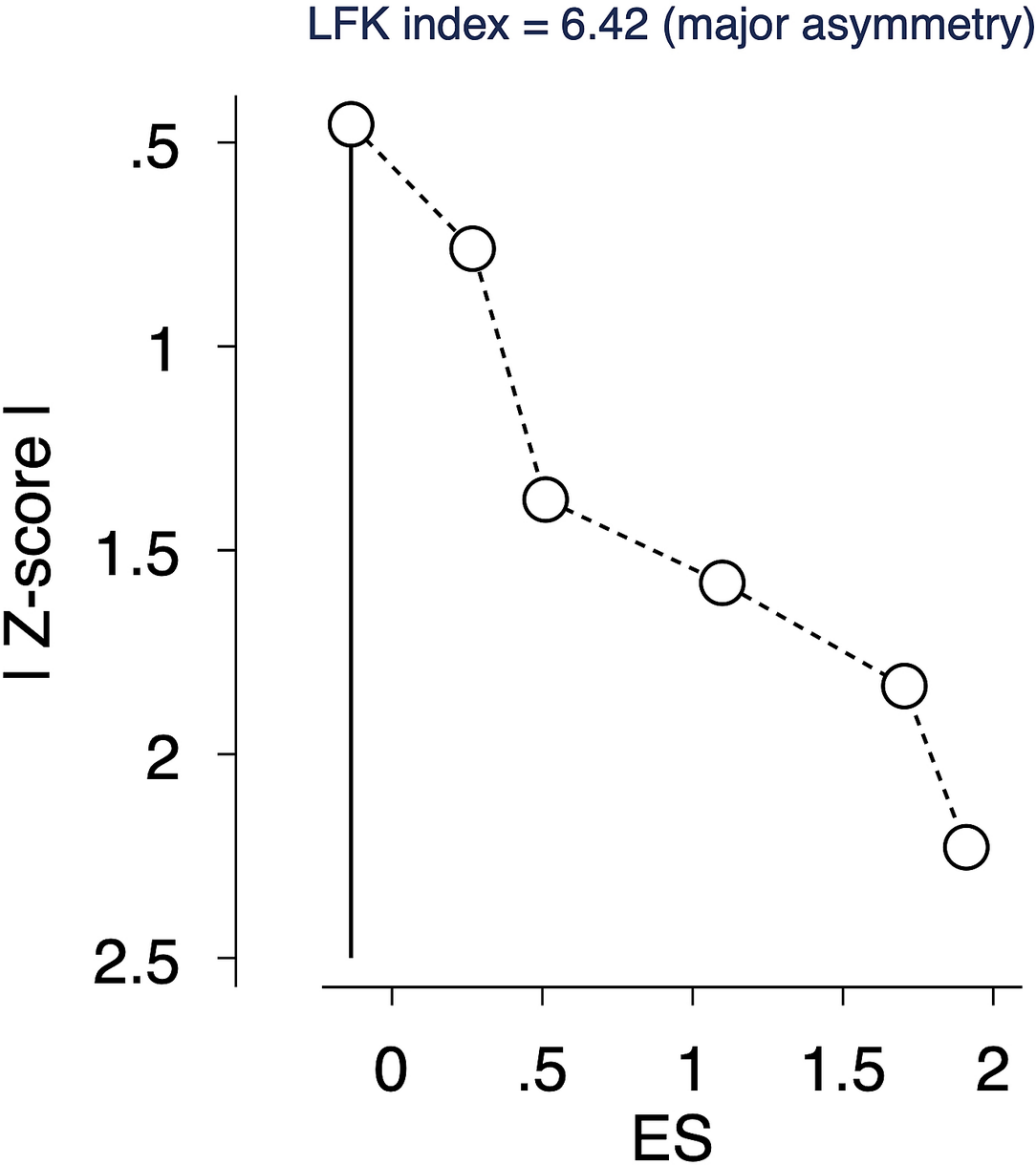
Doi plot for assessing the publication bias.

The LFK index of 6.42 suggested a major asymmetry, indicative of a potential publication bias or other small-study effects (Figure 4).

#### Certainty of evidence

The GRADE assessment of evidence certainty is provided in Table 3.

**Table 3:** Grade assessment.

Certainty assessment							Certainty
№ of studies	Study design	Risk of bias	Inconsistency	Indirectness	Imprecision	Other considerations	
6	randomized and non-randomized trials	serious^a^	very serious^b^	not serious^c^	serious^d^	serious^e^	⨁◯◯◯ Very Low

a High risk of bias in fewer studies.

b Substantial heterogeneity.

c No indirectness found in the parameters.

d Confidence interval is broad.

e Significant publication bias.

Risk of Bias: There was a mixed levels of bias risk across studies, with some studies having high risk and others low or some concerns, suggesting an initial downgrade in the quality of evidence.

Inconsistency: The high degree of heterogeneity (I^2^ = 93.7%, *p* < 0.0001) suggests significant inconsistency across studies, which may lead to a further downgrade in evidence quality.

Indirectness: We found that the studies directly address the research question and populations, interventions, and outcomes as applicable, this domain has not led to a downgrade.

Imprecision: The wide confidence intervals in some study estimates could indicate imprecision, potentially leading to a downgrade depending on the overlap and the width of these intervals.

Publication Bias: The LFK index of 6.42 points to substantial publication bias or small study effects, necessitating a downgrade in the quality of evidence.

Given these considerations, the GRADE assessment for the overall quality of evidence on the effectiveness of financial incentives for hepatitis B vaccination among substance users has been classified as very low.

## Discussion

Our meta-analysis, incorporating six studies with a total of 3,886 participants, revealed a significant effect of financial incentives on hepatitis B vaccination uptake in substance users, with an overall pooled RR of 2.261 (95% CI: 1.327 to 3.851). This finding underscores the potential of financial incentives to substantially enhance vaccination rates in this high-risk group. However, a considerable heterogeneity and a significant LFK index suggest substantial variability among study outcomes and potential publication bias or small-study effects. The GRADE assessment resulted in a very low quality of evidence due to concerns regarding risk of bias, inconsistency, imprecision, and publication bias.

Our findings align with the broader literature, which suggests that financial incentives can be effective in promoting health-related behaviors among high-risk populations, such as substance users (26–28). Previous studies have indicated that financial incentives were effective in increasing rates of screening, vaccination, and treatment adherence for various health conditions (26–30). However, the degree of effectiveness reported in our study exceeds some prior estimates, highlighting the specific efficacy of financial incentives in HBV vaccination uptake. The significant heterogeneity observed in our analysis is consistent with previous meta-analyses in similar fields. We may speculate that this heterogeneity is due to the variability in how financial incentives are implemented and their impact across different settings and populations.

Our analysis offers critical insights into the scalability of financial incentives as a public health intervention. By comparing our findings with existing literature, we can infer that the effectiveness of such incentives may vary not only by demographic factors but also by the nature of healthcare systems and societal norms across different regions (26–30). This variation underscores the need for tailored approaches in implementing financial incentives, suggesting that a one-size-fits-all strategy may not be universally effective. Together with previous research, our results imply that the success of financial incentives hinges on the perceived value of the incentive by the target population, indicating the importance of cultural and economic contexts in shaping responses to such interventions.

The effectiveness of financial incentives can be attributed to several factors. Behavioral economic theory suggests that immediate rewards can significantly influence health behaviors, making financial incentives a potent tool for encouraging vaccination uptake among substance users, who may face barriers to accessing healthcare services (31). The variation in effectiveness across studies could be due to differences in the size of incentives, the method of delivery, or the contextual factors unique to each study’s setting.

This variability emphasizes the complexity of human behavior in health-related decision-making. The decision to accept vaccination, influenced by financial incentives, may be affected by factors such as individual health beliefs, perceived susceptibility to the disease, and trust in medical institutions (32). While financial incentives may address the immediate barriers of access and motivation, they still need to be part of a broader strategy that includes education and outreach to account for these deeper, underlying factors (10). This is particularly important for designing interventions that are not only effective but also sustainable in promoting health behavior change over the long term.

Our study’s primary strength lies in its comprehensive approach. We included a wide range of studies and a substantial participant pool, which provides a robust analysis of the effectiveness of financial incentives on HBV vaccination rates. Additionally, the use of GRADE methodology enhances the reliability of our evidence quality assessment.

However, there are several limitations. The very low quality of evidence, as determined by GRADE, reflects significant concerns about risk of bias, heterogeneity, imprecision, and potential publication bias. The high I^2^ value indicates considerable variability in the study outcomes, which could limit the generalizability of our findings. Moreover, the presence of publication bias, suggested by the LFK index, may have influenced the overall effect size, potentially overstating the effectiveness of financial incentives.

Despite these limitations, our findings have important implications for public health policy and practices. They suggest that financial incentives could be a valuable tool in increasing HBV vaccination rates in substance users, a group traditionally hard to reach with conventional public health interventions. Implementing financial incentives in targeted vaccination campaigns could, thus, contribute to reducing the prevalence of HBV and its associated health burdens in this vulnerable population.

Moreover, the potential of financial incentives to make a significant impact on public health extends beyond HBV vaccination to other areas where behavioral change is crucial for disease prevention and health promotion. For instance, financial incentives may provide substantial public health benefits in populations, affected by the current opioid epidemic and associated health complications, including hepatitis C and HIV. This strategy would contribute to a more holistic approach to managing health risks among substance-using populations, emphasizing the need for integrated healthcare solutions that address a range of interrelated health issues.

Future research should aim to address the limitations identified in this study. Specifically, there is a need for high-quality RCTs with rigorous design and reporting standards to minimize bias and improve the precision of effect estimates. Studies should also explore the impact of different incentive structures and amounts on vaccination uptake to identify the most cost-effective strategies. Additionally, research should focus on understanding the mechanisms through which financial incentives influence behavior change among substance users and the potential long-term effects on HBV prevalence and health outcomes in this population. Cost-effectiveness studies should aim to determine whether the short-term financial outlay associated with incentive programs yields long-term savings in healthcare costs through the prevention of disease. This economic perspective is crucial for policymakers and public health officials in allocating resources effectively to combat public health challenges. As we advance, integrating behavioral economic principles with epidemiological research could revolutionize our approach to disease prevention, particularly in hard-to-reach populations where traditional public health strategies have been less effective.

## Conclusion

Our meta-analysis indicates that financial incentives significantly increase HBV vaccination rates in substance users. Although the evidence in this study is of very low quality due to factors such as heterogeneity, and publication bias, financial incentives still present a promising strategy for public health interventions aimed at increasing vaccination coverage in high-risk populations. More rigorous research is needed to confirm our findings, determine the most effective incentive strategies, and ensure that such interventions can be efficiently integrated into broader public health programs.

## Data Availability

The original contributions presented in the study are included in the article/Supplementary material, further inquiries can be directed to the corresponding author.
