# Supplementary material for: Effectiveness of financial incentives for control of viral hepatitis among substance users: a systematic review and meta-analysis
Source: Front Public Health. 2024 Nov 14;12:1394164. doi: 10.3389/fpubh.2024.1394164 (PMC11602491; doi:10.3389/fpubh.2024.1394164)
Supplement: Supplementary file 1 [file Data_Sheet_1.PDF]

Supplementary File

Search Strategy

Medline Ovid

1. ("Hepatitis B" OR "HBV" OR "Hepatitis B virus")/exp OR "Hepatitis B"[MeSH Terms]

2. ("financial incentives" OR cash OR economic OR financial OR monetary OR payment* OR reward* OR grant* OR award* OR benefit* OR incentive* OR voucher* OR stipend*)

3. ("Conditional Cash Transfer" OR "Cash Incentive" OR "Monetary Incentive")

4. ("Randomized Controlled Trial"[MeSH Terms] OR "Controlled Clinical Trial"[MeSH Terms] OR "RCT" OR "Randomized trial" OR "clinical trial" OR "trial" OR "experiment" OR "study" OR "control group" OR "comparison group")

5. ("Non-randomized trial" OR "quasi-experimental" OR "observational study" OR "cohort study" OR "case-control study" OR "cross-sectional study")

6. 1 AND (2 OR 3) AND (4 OR 5)

7. Limit 6 to (English language AND humans AND yr="1956-2024")

Scopus

(TITLE-ABS-KEY("Hepatitis B" OR "HBV" OR "Hepatitis B virus"))

AND (TITLE-ABS-KEY("financial incentives" OR cash OR economic OR financial OR monetary OR payment* OR reward* OR grant* OR award* OR benefit* OR incentive* OR voucher* OR stipend*))

AND (TITLE-ABS-KEY("Conditional Cash Transfer" OR "Cash Incentive" OR "Monetary Incentive"))

AND (TITLE-ABS-KEY("Randomized Controlled Trial" OR "clinical trial" OR "trial" OR "experiment" OR "study" OR "control group" OR "comparison group" OR "Non-randomized trial"

OR "quasi-experimental" OR "observational study" OR "cohort study" OR "case-control study" OR "cross-sectional study"))

AND (LIMIT-TO(LANGUAGE, "English") AND LIMIT-TO(DOCTYPE, "ar"))

EMBASE

1. 'hepatitis b'/exp OR "Hepatitis B virus":ab,ti OR "HBV":ab,ti
2. 'financial incentives'/exp OR (cash OR economic OR financial OR monetary OR payment* OR reward* OR grant* OR award* OR benefit* OR incentive* OR voucher* OR stipend*):ab,ti
3. ('Conditional Cash Transfer' OR 'Cash Incentive' OR 'Monetary Incentive'):ab,ti
4. 'randomized controlled trial'/exp OR 'clinical trial'/exp OR 'trial':ab,ti OR 'study':ab,ti OR 'experiment':ab,ti OR 'control group':ab,ti OR 'comparison group':ab,ti
5. ('Non-randomized trial' OR 'quasi-experimental' OR 'observational study' OR 'cohort study' OR 'case-control study' OR 'cross-sectional study'):ab,ti
6. 1 AND (2 OR 3) AND (4 OR 5)
7. Limit 6 to (English language AND humans AND yr="1956-2024")

Cochrane CENTRAL

#1 MeSH descriptor: [Hepatitis B] explode all trees OR "Hepatitis B" OR "HBV" OR "Hepatitis B virus"

#2 MeSH descriptor: [Financial Incentives] explode all trees OR (cash OR economic OR financial OR monetary OR payment* OR reward* OR grant* OR award* OR benefit* OR incentive* OR voucher* OR stipend*)

#3 ("Conditional Cash Transfer" OR "Cash Incentive" OR "Monetary Incentive")

#4 "Randomized Controlled Trial" OR MeSH descriptor: [Randomized Controlled Trial] explode all trees OR "clinical trial" OR "trial" OR "experiment" OR "study" OR "control group" OR

"comparison group"

#5 ("Non-randomized trial" OR "quasi-experimental" OR "observational study" OR "cohort study" OR "case-control study" OR "cross-sectional study")

#6 #1 AND (#2 OR #3) AND (#4 OR #5)

#7 Limits: Publication Year from 1980 to 2024, Humans, English
