# Supplementary figures and images for: Effectiveness of financial incentives for control of viral hepatitis among substance users: a systematic review and meta-analysis
Source: Front Public Health. 2024 Nov 14;12:1394164. doi: 10.3389/fpubh.2024.1394164 (PMC11602491; doi:10.3389/fpubh.2024.1394164)

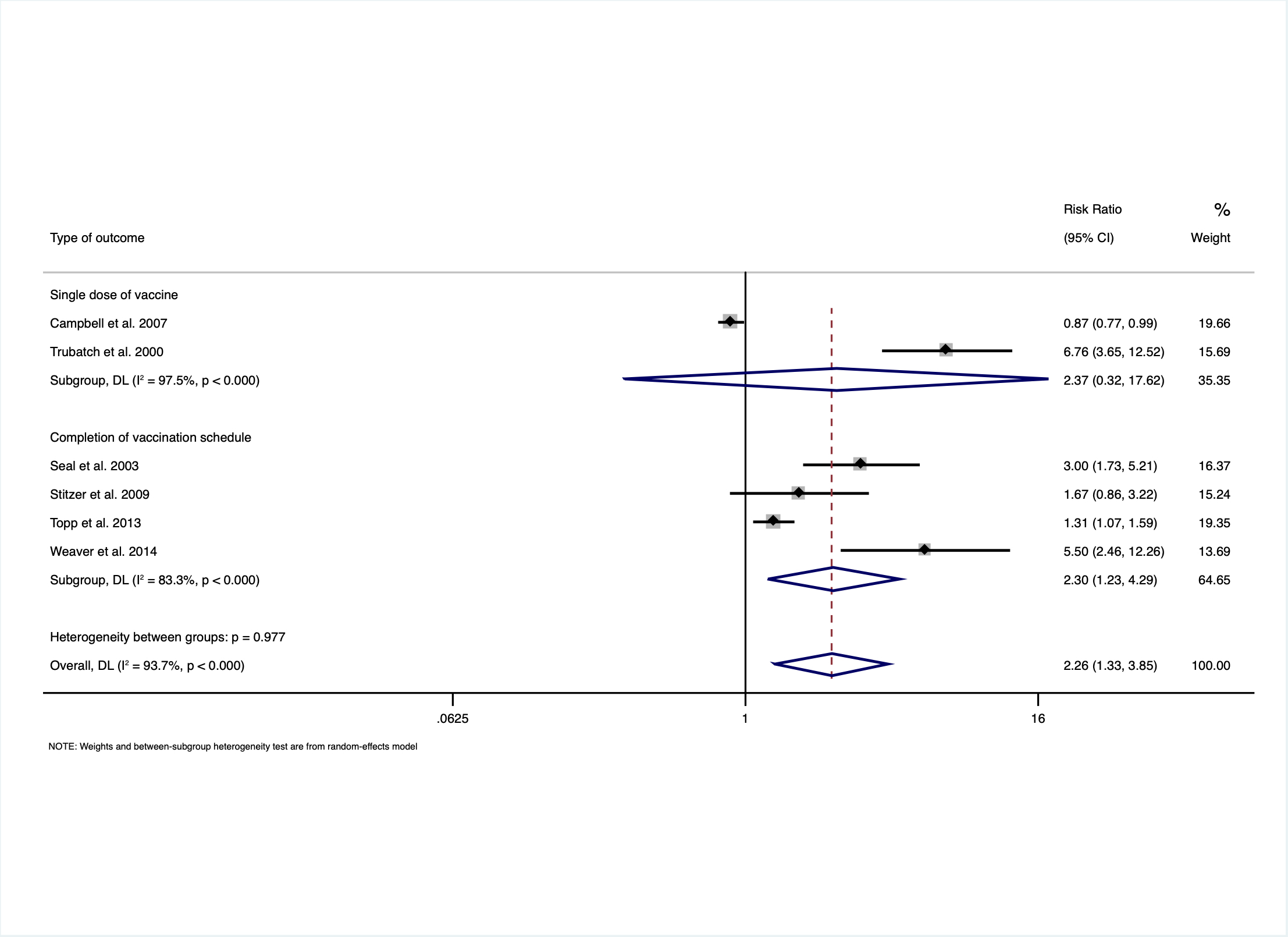

Supplement: Supplementary file 2 [file Image_1.JPEG]

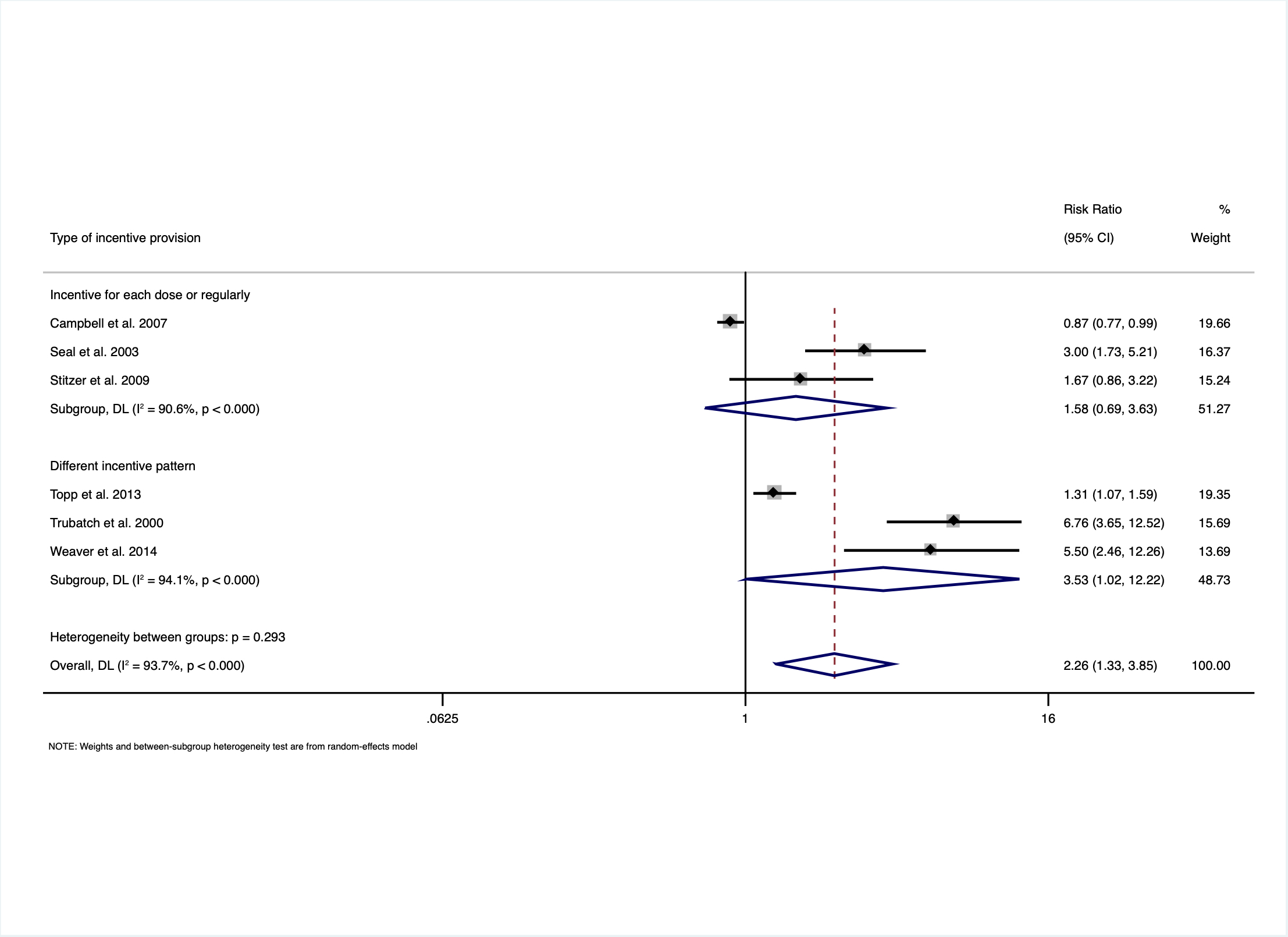

Supplement: Supplementary file 3 [file Image_2.JPEG]
